# Emotional Overshadowing: Pleasant and Unpleasant Cues Overshadow Neutral Cues in Human Associative Learning

**DOI:** 10.1007/s42761-024-00270-0

**Published:** 2024-09-07

**Authors:** Jianming Zhu, Angela Radulescu, Daniel Bennett

**Affiliations:** 1 School of Psychological Sciences, Monash University, Melbourne, Australia; 2 Icahn School of Medicine at Mount Sinai, New York City, USA

**Keywords:** Attention, Associative learning, Emotion, Overshadowing

## Abstract

When learning about stimuli comprised of multiple cues, humans and other animals tend to form stronger cue-outcome associations for more salient cues than for less salient cues. This phenomenon, termed *overshadowing*, has typically been demonstrated between cues that vary in salience because of differences in physical intensity. In this study, we investigated whether differences in the emotional valence of cues in a compound stimulus similarly led to differences in the strength of cue-outcome learning. Using a probabilistic categorisation task in which stimuli were compounds consisting of pairs of emotional or non-emotional cue images*,* we found consistent evidence for emotional overshadowing across both an initial exploratory study (*N* = 50) and a confirmatory preregistered replication study (*N* = 200). Specifically, both pleasant and unpleasant cue images tended to overshadow neutral cue images, but pleasant and unpleasant cue images did not overshadow one another. Moreover, across stimuli, the magnitude of differences in learning between cues was proportional to differences in their absolute emotional valence, suggesting that attentional capture by both positively and negatively valenced emotions drives overshadowing. These findings have implications for understanding associative learning in natural environments, where stimuli are frequently imbued with emotional valence prior to learning.

**Supplementary Information:**

The online version contains supplementary material available at 10.1007/s42761-024-00270-0.

The capacity limitations of human attention mean that some stimuli in the environment are prioritised for cognitive processing over others (Driver, 2001), with more salient stimuli attracting more attention and receiving more extensive cognitive processing than less salient stimuli. Broadly, factors that influence salience include the physical intensity, perceptual distinctiveness, and self- or goal-relevance of a stimulus (for review see Bordalo et al., 2022). In addition, the salience of a stimulus is also modulated by its emotional valence: stimuli with either a positive or a negative emotional valence (i.e., pleasant/rewarding or unpleasant/threatening stimuli) tend to capture attention more than stimuli without strong emotional valence (Yiend, 2010). In the present study, we sought to investigate the implications for associative learning of this attentional prioritisation of emotional stimuli.

In the appetitive domain, it has been shown that irrelevant stimuli associated with reward tend to capture attention during task performance, even when this interferes with performance of a concurrent task (Pearson et al., 2022). Likewise, research in the aversive domain has shown that negatively valenced images—such as unpleasant and threatening stimuli—tend to automatically attract attention, particularly for individuals with high levels of anxiety (MacLeod & Mathews, 1988; Mogg & Bradley, 2018). The interactions between attention and emotion have been shown to play out across multiple task domains (Most et al., 2005; Öhman et al., 2001; Yiend, 2010); however, relatively little work to date has explored how emotion-attention interactions might shape associative learning (Bennett et al., 2023).

In associative learning, a long history of research with non-emotional stimuli has shown that competition for attention between different cues gives rise to a phenomenon termed *overshadowing* (Pavlov, 1927), in which observers tend to learn stronger cue-outcome associations for the more salient of two cues in a compound. A paradigmatic example is given by Mackintosh (1976), who trained rats to associate a compound comprised of two cues (a light and an auditory tone) with an aversive unconditioned stimulus (a foot shock). By varying the physical intensity of the tone (and therefore its salience) during conditioning, Mackintosh showed that the salience of the tone affected the degree to which rats learned to associate it with shock. Specifically, when the tone was loud, animals primarily associated the shock with the tone rather than the light (i.e., the loud tone *overshadowed* the light), but primarily associated the shock with the light when the tone was quiet (i.e., the light overshadowed the quiet tone). Attentional models of this effect (e.g., Mackintosh, 1975) explain overshadowing by proposing that animals learn about different cues in proportion to the degree of attention allocated to each (though non-attentional models of overshadowing have also been proposed; see Rescorla & Wagner, 1972). Studies in human participants have also shown evidence for attention-related effects in associative learning for cues varying in salience (Alcalá et al., 2023; Byrom & Murphy, 2019; Le Pelley et al., 2014), including for compounds containing salient social stimuli (happy and angry faces; Lanzetta & Orr, 1980, 1981).

When taken together, these two lines of research on attentional prioritisation of emotional stimuli and on overshadowing in associative learning give rise to a hitherto untested prediction: that emotional stimuli (both pleasant and unpleasant) will overshadow non-emotional stimuli in human associative learning. The logic of this prediction is that when participants learn stimulus-outcome contingencies for a compound stimulus comprised of cues that vary in emotional valence, attentional prioritisation of emotional cues in the compound means that participants should form stronger cue-outcome associations for emotionally valenced cues than for emotionally neutral cues. In other words, we would expect *emotional overshadowing* to take place. Across two experiments, the present study tested this prediction among human participants completing a novel associative learning task in which emotionally valenced images from the OASIS stimulus set (Kurdi et al., 2017) were used as cues within compound conditioned stimuli.

## Method

### Design, Preregistration, and Data Availability

This study comprised an initial exploratory study, followed by a preregistered confirmatory replication study. Since the two studies used an identical experimental design, we present the method and results for both studies side-by-side below. All data analysed in this study, along with the preregistration document for the replication study, are publicly available in an Open Science Framework repository at https://osf.io/q5x6f/. There were no deviations from the preregistered analysis plan in the replication study, with the exception of an exploratory joint regression analysis (of combined data from both studies) that we ran as a follow-up to the preregistered analyses.

### Participants

Participants were adults aged 18 to 65 who resided in Australia, Canada, Ireland, New Zealand, and the UK, and were recruited via the website Prolific. We initially recruited 50 participants for the initial exploratory study and 200 participants for the preregistered replication; after applying exclusion criteria (see below), the initial exploratory sample comprised 44 participants (*M*_*age*_ = 34.4 years; 18 men, 23 women, 3 who did not endorse a binary gender), and the replication sample comprised 168 participants (*M*_*age*_ = 36.2 years; 98 men, 70 women).

Total time commitment was approximately 25 min, and participants received a base payment of USD $5 plus a bonus up to $1 depending on task performance (initial exploratory study: *M* = $0.62; preregistered replication: *M* = $0.73). Both studies were approved by the Monash University Human Research Ethics Committee (#32,391) and participants provided informed consent via web browser.

The sample size for the replication study was determined using a simulation-based bootstrap power analysis based on the results of the initial exploratory study. Specifically, we estimated the sample size necessary to have in excess of 80% power to detect each of the primary results of the exploratory study by repeatedly resampling participants from the exploratory dataset (with replacement) and simulating new choices for each trial for each resampled participant according to a posterior predictive check for a *brms* regression model. We then re-fitted *brms* models to the resulting synthetic data to determine the proportion of simulated datasets within which the 95% Bayesian Highest Density Interval (HDI) did not include zero and was in the same direction as the effect in the exploratory sample. Further details of this power analysis are available in the preregistration document.

### Materials and Procedure

All study components were presented to participants in their web browser using custom code written in JavaScript using the jsPsych package (De Leeuw, 2015) and Python server code written using the Flask web framework and hosted on a Monash University virtual machine.

#### Questionnaires

Before the behavior task, participants completed a self-report measure of current anxiety symptom severity (the GAD-7; Spitzer et al., 2006), modified to include one infrequency-item attention check. This attention check was used to exclude participants who showed evidence of inattentive survey responding; this was of particular importance given the expected positive skew in the distribution of anxiety symptom scores (see Zorowitz et al., 2023).

#### Probabilistic Categorisation Task

**Fig. 1:**
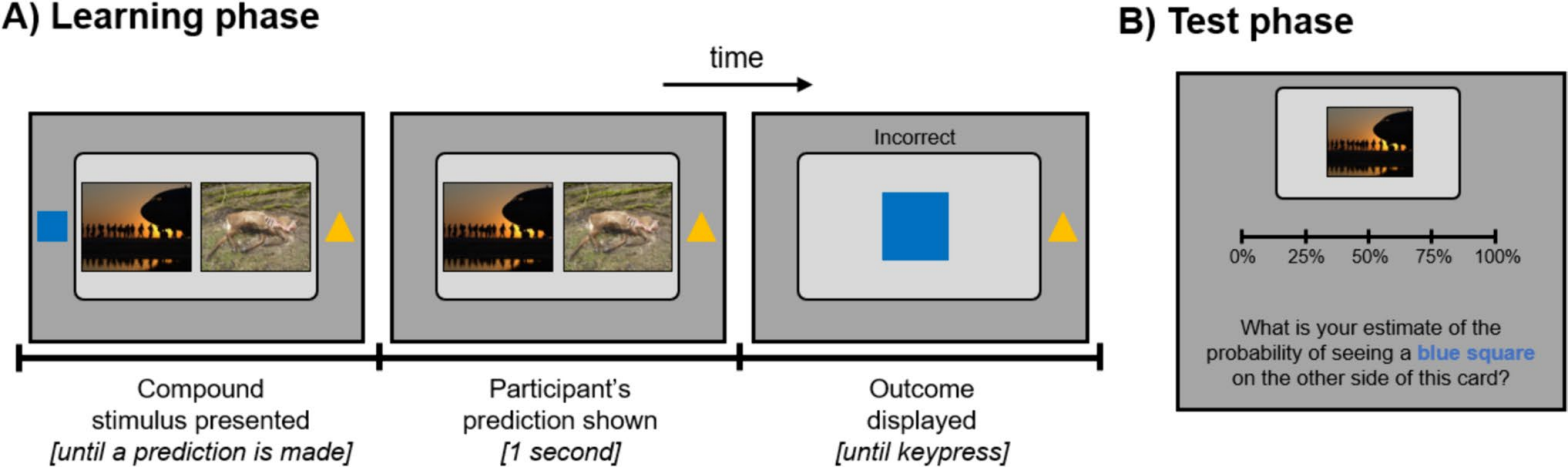
Task schematic for the probabilistic categorisation task. In each trial during the learning phase (**A**), participants used the left/right arrow keys to make a prediction about the shape (either a blue square or a yellow triangle) that would be found on the other side of a compound ‘flashcard’ stimulus composed of two cue images (here, a neutral image on the left and an unpleasant image on the right). The card was then ‘flipped over’ to reveal feedback on the accuracy of their prediction. During the test phase (**B**), participants then used a sliding scale to indicate the probability with which different images were associated with each of the outcome shapes

Participants completed a custom probabilistic categorisation task in which they learned about four ‘flashcard’ compound stimuli, each comprising a pair of emotionally valenced image cues (drawn from the OASIS stimulus set; Kurdi et al., 2017). Each flashcard stimulus was probabilistically associated with one of two outcome shapes (either a blue square or a yellow triangle). Participants first completed a *learning phase* (Fig. 1A), in each trial of which they were shown a flashcard stimulus and asked to predict what shape was on the other side of the flashcard. They then received probabilistic feedback: each stimulus was associated with one outcome shape on 80% of trials and the other shape on the remaining 20%. Across three learning blocks of 42 trials each, participants were exposed to each of the four stimuli 30 times (trial order randomised), plus six attention-check trials in which participants were simply shown one of the two outcome shapes and asked to report which shape it was. Participants who responded inaccurately to any attention-check trials were excluded from all further analyses.

Crucially, the four flashcard stimuli about which participants learned each contained different pairs of emotionally valenced cue images. Each participant learned about (a) a *pleasant/neutral* flashcard comprising one pleasant image cue and one neutral image cue, (b) an *unpleasant/neutral* flashcard (one unpleasant image, one neutral image), (c) a *pleasant/unpleasant* flashcard (one pleasant image, one unpleasant image), and (d) a *neutral/neutral* flashcard (two neutral images). The images within each flashcard stimulus were unique, and so each participant effectively learned about eight distinct cue images across the four flashcard stimuli. The allocation of images to stimuli was randomised, as was the left/right mapping of images within a flashcard stimulus on each trial. The mapping of stimuli to shape outcomes was pseudo-randomised for each participant, subject to the constraint that ‘yellow triangle’ was the correct prediction for two of the four flashcards and ‘blue square’ was the correct prediction for the other two flashcards. Prior to completing the behavior task, participants provided ratings of valence (i.e., pleasantness/unpleasantness) and arousal (i.e., emotional intensity) for each of the eight cue images.^1^

In the *test phase* of the task, participants were asked to estimate the probabilistic contingencies between each of the eight single-image cues and each of the two outcome shapes (Fig. 1B; participants also made probability estimates for the four original compound stimuli). From these data, overshadowing can be measured as the difference in the estimated outcome contingencies between the two images that were conditioned together within the same compound (e.g., if emotional overshadowing occurs within the pleasant/neutral stimulus, we would expect participants to be more confident in their contingency reports for the pleasant cue and less confident for the neutral cue). In addition, participants also made several explicit category predictions for each of the image cues (without feedback).

### Data Analysis

For each image, participants gave probability reports for both outcome shapes (i.e., both the ‘correct’ shape and the ‘incorrect’ shape). To make probability reports directly comparable across images and outcomes, probability reports were transformed into a confidence statistic:

$$\text{Confidence}=\frac{\text{abs}(\text{Reported probability}-50)-30}{50}$$

This procedure transforms probability ratings onto a scale in which 0 indicates perfectly calibrated probability reports, positive values indicate overconfident probability reports (e.g., reporting 90% probability when the true contingency was 80% or 10% when the true contingency was 20%), and negative values indicate underconfident probability reports.

Data were analysed separately for each stimulus using a Bayesian mixed-effects regression as implemented in the *brms* package (Bürkner, 2017) for R (see Supplementary Material for further details of regression models). Effects were taken to be credibly different from 0 if then 95% Bayesian HDI did not include zero.

## Results

### Image Ratings

**Fig. 2:**
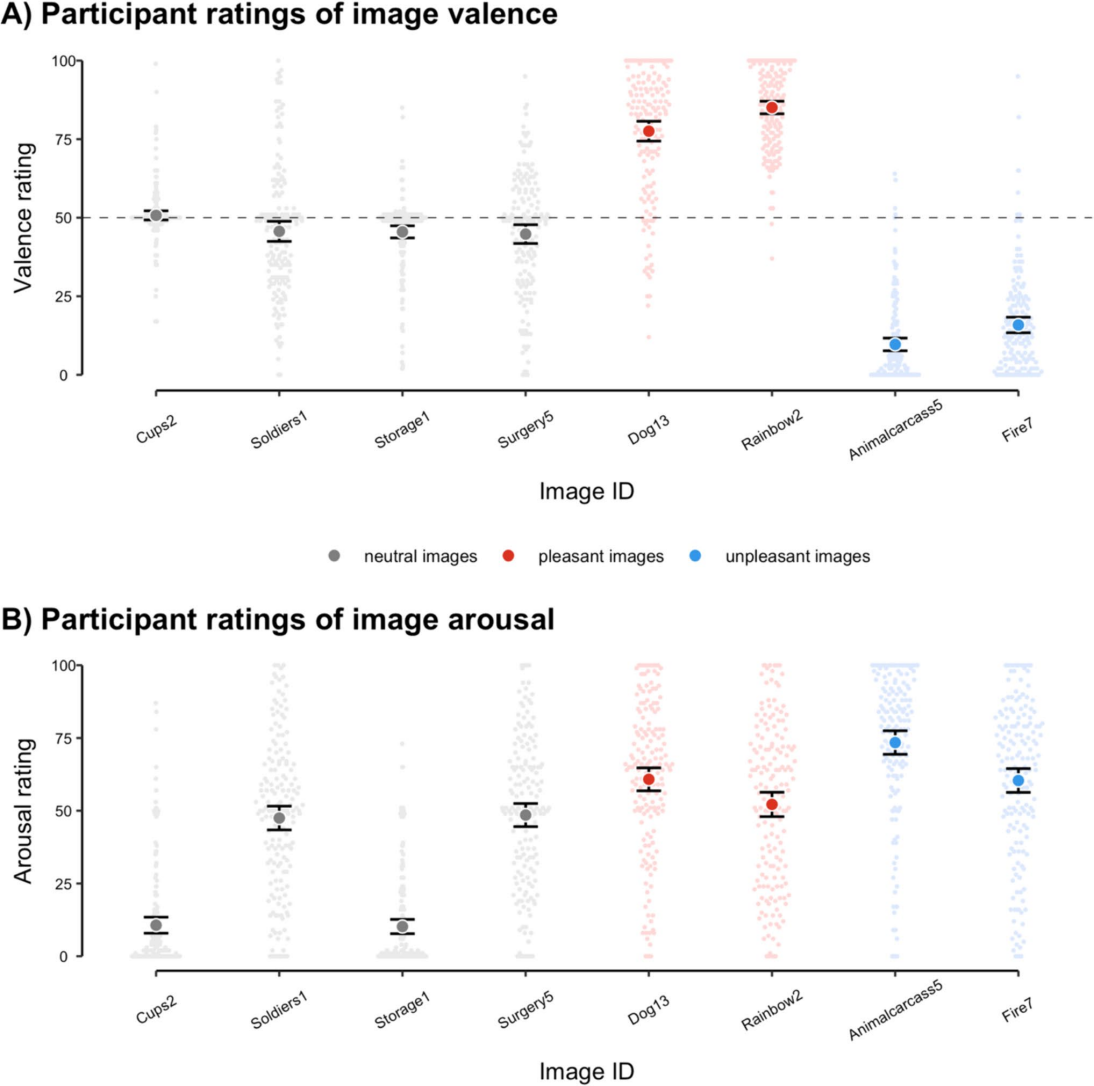
Participant ratings of valence (**A**) and arousal (**B**) for each image in each category (grey, neutral-valence images; red, pleasant-valence images; blue, unpleasant-valence images) in the preregistered replication study. Foreground points and error bars denote group means (± 95% confidence interval); background points denote ratings from individual participants. The dashed horizontal line in (A) denotes neutral valence (‘neither positive nor negative’)

We first conducted a manipulation check of participants’ image ratings. As expected, pleasant (/unpleasant) images were rated as substantially more positive (/negative) in valence than neutral images. Similarly, neutral images were rated as lower in arousal compared with both pleasant and unpleasant images (see Supplementary Tables S1 and S2 for full regression coefficient estimates). For both valence and arousal, there were considerable individual differences in ratings for each image (see Fig. 2 for image ratings in the replication study and Supplementary Figure S1 for ratings in the initial exploratory study). In subsequent regression analyses, we therefore used each participant’s own ratings of valence and arousal for each image as predictors.

### Learning Phase

**Fig. 3:**
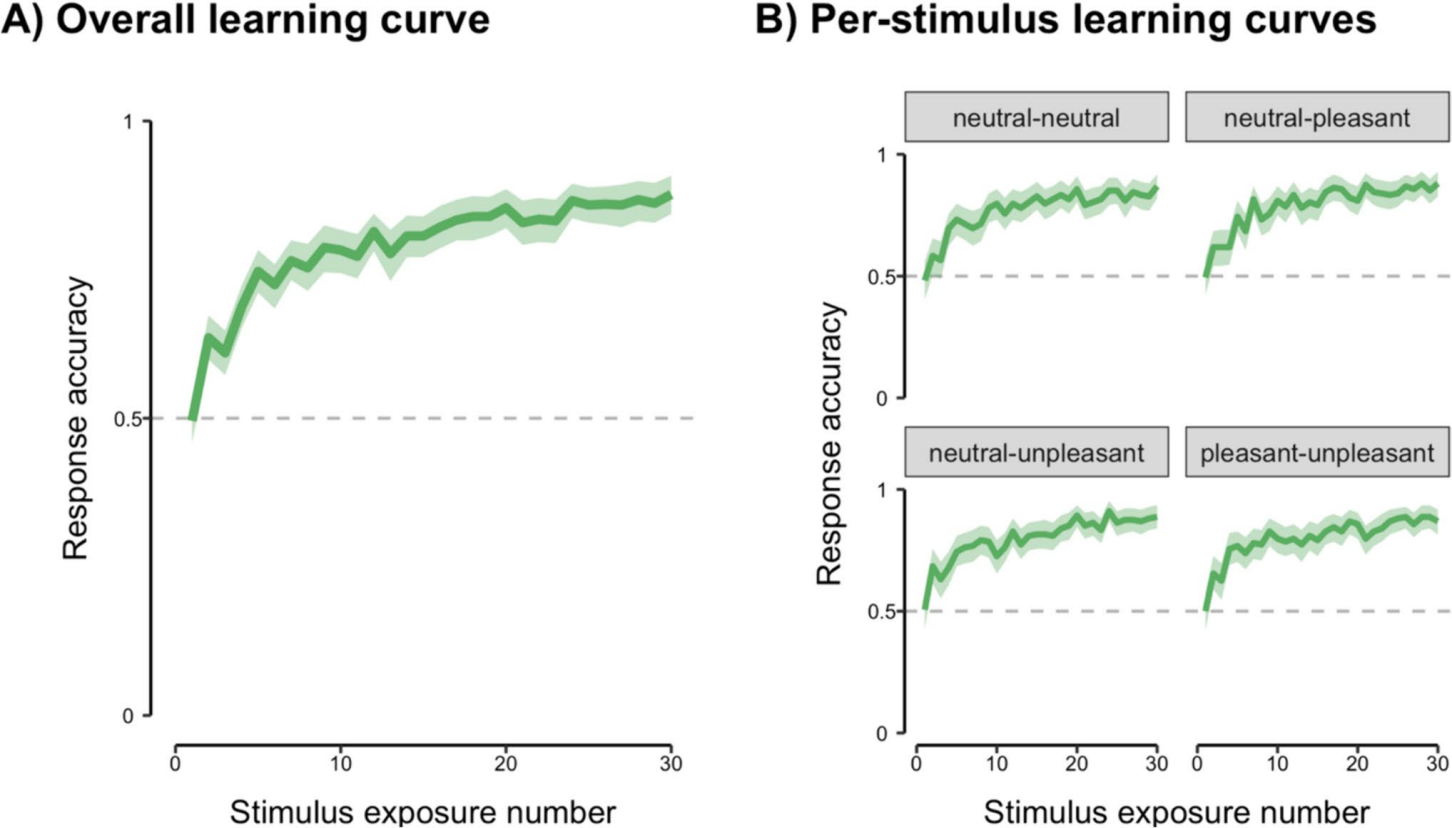
Response accuracy as a function of stimulus exposure number during the learning phase of the probabilistic categorisation task for the preregistered replication study. Results are presented overall (**A**) and individually by stimulus type (**B**). Data are mean response accuracy (with a correct response defined as predicting the most likely outcome for a stimulus) ± 95% confidence interval. The dashed horizontal line denotes chance-level accuracy

Participants successfully learned stimulus-outcome contingencies to asymptote (mean prediction accuracy in the final block of the learning phase was 78.4% in the exploratory study and 85.4% in the replication study; see Fig. 3 and Supplementary Figure S2, respectively) In the replication study (but not the initial exploratory study), a Bayesian mixed-effects logistic regression analysis also found evidence for an interaction between card type and response accuracy, driven by lower mean accuracy and slower learning rate for the neutral/neutral stimulus; see Supplementary Table S3 for full regression coefficient estimates.

### Emotional Overshadowing

**Fig. 4:**
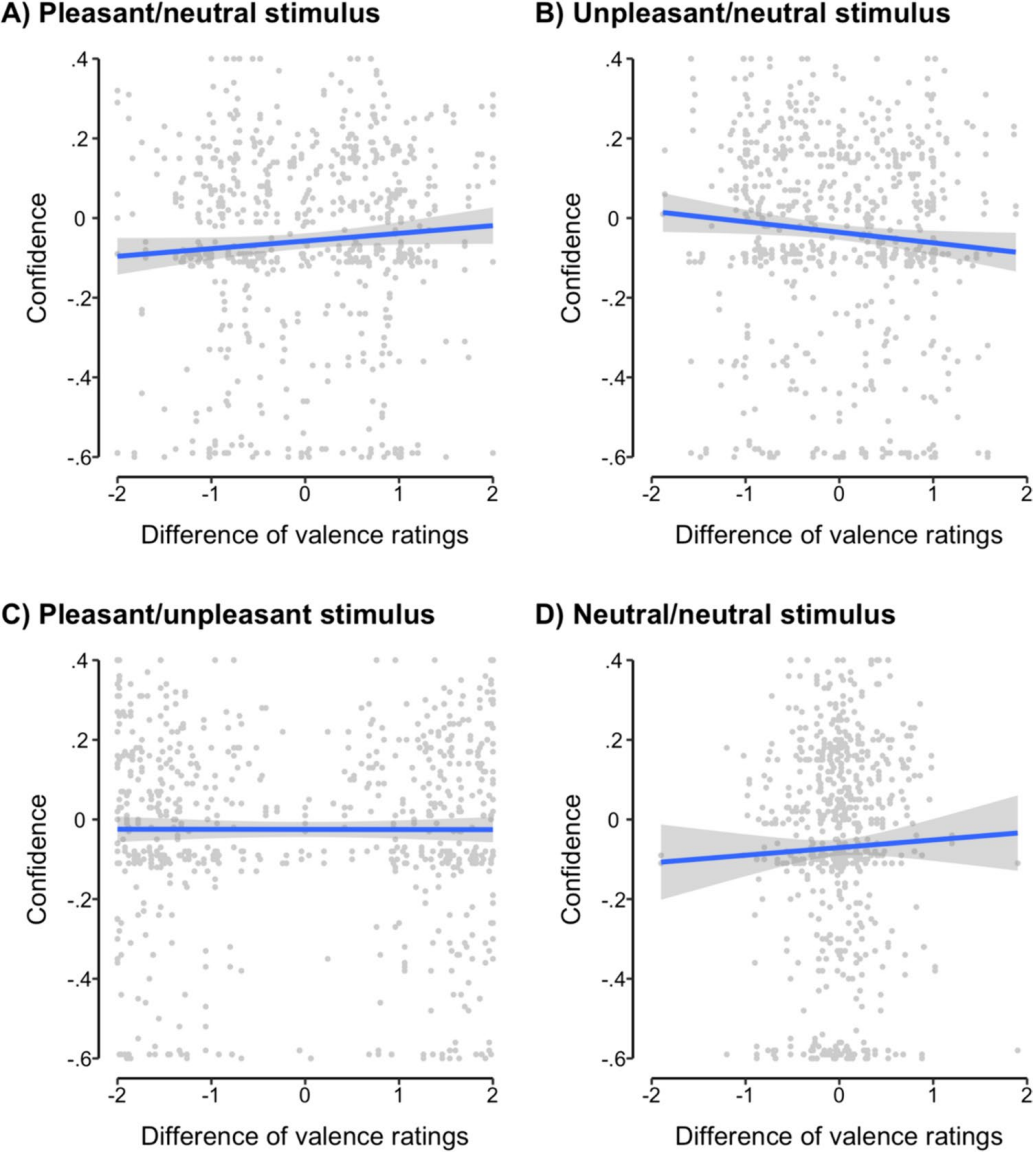
The association between the difference in valence ratings between the two images in a stimulus (*x*-axis) and participants’ confidence regarding reported cue-outcome associations (*y*-axis). There was a significant positive association between valence difference and confidence for the pleasant/neutral stimulus (**A**), a significant negative association for the unpleasant/neutral stimulus (**B**), and no significant association for either the pleasant/unpleasant stimulus (**C**) or the neutral/neutral stimulus (**D**). Regression lines indicate the linear association of best fit and its 95% confidence interval. Each background point represents one self-report by one participant. Data are presented for the preregistered replication study only

For each stimulus, we tested for the presence of emotional overshadowing by analysing the effects of image valence on the strength of reported cue-outcome associations. A full description of regression models and coefficient estimates is presented in Supplementary Tables S4-S7; valence results are visualised in Fig. 4 for the replication study and Supplementary Figure S3 for the initial exploratory study.

For cue images within the pleasant/neutral stimulus, we consistently found a significant positive effect of self-reported image valence on the strength of cue-outcome associations (exploratory study, *β*_*valence*_ = 0.06, 95% HDI = [0.02, 0.10]; replication study, *β*_*valence*_ = 0.02, 95% HDI = [0.004, 0.04]). This indicates that within this stimulus, participants formed stronger associations for more pleasant images and weaker associations for less pleasant images: in other words, emotional overshadowing occurred. In the initial exploratory study, we also found that this effect of valence was moderated by individual differences in self-reported anxiety (*β* = 0.04, 95% HDI = [0.001, 0.08]), although this was not replicated in the replication study (*β* = 0.003, 95% HDI = [− 0.01, 0.02]).

Similarly, we also found evidence of emotional overshadowing for cue images within the unpleasant/neutral stimulus. For this stimulus, we found a *negative* effect of stimulus valence (exploratory study, *β*_*valence*_ = − 0.07, 95% HDI = [− 0.13, − 0.01]; replication study, *β*_*valence*_ = − 0.03, 95% HDI = [− 0.06, − 0.001]). In other words, for cues within the unpleasant/neutral stimulus, participants tended to form stronger cue-outcome associations for the more unpleasant images and weaker cue-outcome associations for the less unpleasant images. This effect was not significantly moderated by anxiety.

We did not find evidence of emotional overshadowing for cues within either the pleasant/unpleasant stimulus (exploratory study, *β*_*valence*_ = 0.004, 95% HDI = [− 0.01, 0.02]; replication study, *β*_*valence*_ = 0.01, 95% HDI = [− 0.001, 0.01]) or the neutral/neutral stimulus (exploratory study, *β*_*valence*_ = − 0.05, 95% HDI = [− 0.14, 0.03]; replication study, *β*_*valence*_ = 0.02, 95% HDI = [− 0.01, 0.06]). We found a moderating effect of anxiety on emotional overshadowing for the pleasant/unpleasant stimulus in the initial exploratory study (*β* = 0.03, 95% HDI = [0.01, 0.05]); once again, however, this moderation effect did not replicate in the replication study (*β* = 0.004, 95% HDI = [− 0.01, 0.003]). There were no moderating effects of anxiety on emotional overshadowing in the neutral/neutral stimulus.

### Exploratory Joint Analyses

In the stimulus-by-stimulus analyses above, we found evidence for overshadowing when one cue image was emotionally valenced and the other cue image was neutral (i.e., in pleasant/neutral stimuli and unpleasant/neutral stimuli). By contrast, we did not find evidence of overshadowing in pleasant/unpleasant or neutral/neutral stimuli. One interpretation of these findings is that overshadowing may specifically occur when there are differences in the absolute emotional valence in a compound stimulus (i.e., when one cue has emotional valence and the other does not; calculated as $abs(valence-50)$ when valence is measured on a 0–100 scale with 50 as the neutral midpoint). As an exploratory non-preregistered follow-up, therefore, we tested this possibility in a joint regression of the aggregate behavior dataset collapsed across experiments. Results indicated that there was indeed a significant overall effect of the difference in *absolute* valence between the two cues in a stimulus on the strength of cue-outcome associations (see Table 1). In other words, across all stimuli, overshadowing tended to occur when one cue was substantially more emotionally valenced in *absolute* terms than the cues with which it was paired during learning (regression analyses conducted separately in each of the two analyses produced equivalent results; see Supplementary Table S8).

**Table 1:** Coefficients for aggregate regression analysis of emotional overshadowing

Coefficient	*β* (SE)	95% Bayesian HDI	
Intercept	− 0.04 (0.01)	[− 0.05, − 0.02]	*
Queried shape	0.01 (0.01)	[− 0.003, 0.02]	
Confidence rating for corresponding compound stimulus	0.51 (0.03)	[0.45, 0.57]	*
Anxiety	− 0.01 (0.01)	[− 0.02, 0.01]	
Valence difference between cues in compound	− 0.0001 (0.003)	[− 0.01, 0.01]	
Arousal difference between cues in compound	0.002 (0.01)	[− 0.01, 0.01]	
Difference in absolute valence between cues	0.03 (0.01)	[0.01, 0.05]	*
Anxiety $\times$ valence difference	0.004 (0.003)	[− 0.002, 0.01]	
Anxiety $\times$ arousal difference	0.0001 (0.01)	[− 0.01, 0.01]	
Anxiety $\times$ difference in absolute valence	0.01 (0.01)	[− 0.01, 0.03]	

The asterisk (*) denotes coefficients for which the Bayesian 95% HDI excludes zero

This joint regression analysis also revealed a significantly negative intercept (indicating that, on average across all stimuli, participants were slightly underconfident) and a significant effect of the confidence rating for the corresponding compound stimulus on confidence ratings for each individual image. This latter result indicates that, all else being equal, participants tended to have greater confidence about an individual image if they also had greater confidence about the compound image within which they first learned about the individual image.

## Discussion

In this study, we sought to characterise how attentional biases towards emotional stimuli influenced human associative learning. Two experiments (one exploratory, one preregistered replication) consistently provided evidence for a phenomenon that we term *emotional overshadowing*: participants were presented with compound stimuli in which there was an imbalance in the absolute emotional valence of cues (i.e., a compound consisting of a pleasant image and a neutral image, or of an unpleasant image and a neutral image) formed stronger cue-outcome associations for the emotional image and weaker associations for the non-emotional image. We found evidence for this effect across both positive and negatively valenced cues, with pleasant and unpleasant images both overshadowing neutral images during associative learning.

These results are reminiscent of the classical overshadowing effect, in which a physically salient cue (such as a bright light) forms stronger associations with an unconditioned stimulus than a less salient cue (such as a quiet auditory tone) presented concurrently (Pavlov, 1927). Under attentional models of overshadowing (e.g., Mackintosh, 1975, 1976), this result is explained by greater attentional capture, and therefore more rapid learning, for more physically intense cues. Viewed from this perspective, our findings suggest that more emotional cues attracted greater attention—and therefore entered into stronger associations with outcomes—during the learning phase of the task. Moreover, because the effect of stimulus valence on learning was in opposite directions in pleasant/neutral compound stimuli compared with unpleasant/neutral compound stimuli, we can conclude that attention was guided during learning by the *absolute* emotional valence of cue images, rather than by either pleasantness or unpleasantness alone. In further support of this conclusion, we did not see evidence of overshadowing within neutral/neutral compounds (two cues lacking in emotional valence) or within pleasant/unpleasant compounds (two cues with opposing emotional valence). The lack of emotional overshadowing in the pleasant/unpleasant compound, in particular, aligns with results reported by Mackintosh (1976), who found that when rats were presented with cues of equal salience, overshadowing did not occur when the overall intensity of both cues was high.

When we analysed all data simultaneously in an exploratory joint regression analysis, we found that learning strength was significantly predicted by differences in absolute valence between the cue images in a compound even after accounting for cue-level differences in (signed) valence and arousal. Indeed, across all analyses, we included image arousal as a regressor, but consistently found no association between arousal ratings and the strength of cue-outcome learning. This suggests that the effects we observed were related to the perceived valence of images, independent of their overall emotional intensity.

Although these effects are consistent with the proposition that emotional valence influences the associability of cues during learning, they are partly inconsistent with previous results reported in the aversive domain. In a shock-conditioning paradigm, Lanzetta and Orr (1980, 1981) found that angry face stimuli overshadowed a neutral tone stimulus (in line with our findings), but that a neutral tone overshadowed happy face stimuli. The latter result is inconsistent with our finding that pleasant cues overshadowed neutral cues, and further research is therefore required to determine how our results generalise to cases in which the unconditioned stimulus is aversive (rather than neutral, as in the present study).

We also assessed the moderating effects of anxiety on emotional overshadowing. Cognitive models link anxiety with attentional capture by emotional stimuli (threatening stimuli in particular; MacLeod & Mathews, 1988), and therefore predict stronger emotional overshadowing with more severe anxiety. In this respect, our findings were inconsistent: in the initial exploratory study, anxiety significantly moderated emotional overshadowing for pleasant/neutral and pleasant/unpleasant stimuli, but these effects did not replicate in the replication study. However, with only two probability ratings per image per participant, our behavior task was not designed to reliably measure individual differences, which may have limited our capacity to detect moderation effects by participant-level covariates such as anxiety. Further research on this question should therefore develop a variant of the emotional overshadowing task that is optimised for reliable measurement of individual differences.

A limitation of this study is that we assayed attention indirectly—via probability ratings for cue-outcome contingencies—rather than directly, via eye-tracking. Although common practice in associative learning research (e.g., Lagnado & Shanks, 2002), a limitation of this method is that the link we have drawn between attention and emotion is a theoretically grounded inference rather than an empirical observation. Our experimental design also did not control for differences in low-level visual features of images (e.g., edge density, symmetry) that may be predictive of emotional valence (Redies et al., 2020). We consider it likely that emotional valence was the primary factor driving participants’ attention, especially because regression results showed an effect of individual differences in image valence ratings on emotional overshadowing. However, it remains possible that low-level image properties, rather than emotional valence per se, could also explain our results. Future work could investigate this possibility by testing emotional overshadowing among visually similar cues that have acquired positive or negative valence via pre-learned associations with reward/punishment.

Our findings unite previous work on the links between attention and emotion with research on the role of attention in associative learning. The former body of work has demonstrated interactions between attention and emotion including value-modulated attentional capture (Pearson et al., 2022), emotion-induced blindness (Most et al., 2005), affect-congruent compound generalisation (Bennett et al., 2023), and facilitated visual search for emotional stimuli (Öhman et al., 2001). Here, we unite this perspective with the latter body of work, which has shown evidence of (non-emotional) overshadowing and related attentional phenomena in human associative learning (Alcalá et al., 2023; Byrom & Murphy, 2019; Le Pelley et al., 2014; but see also Murphy & Dunsmoor, 2017). In this way, our results demonstrate that attentional capture by emotional cues has implications for rates of associative learning about those cues. We speculate that emotional overshadowing may be a phenomenon of particular relevance within digital environments that contain a multitude of emotional cues competing for users’ attention, such as social media, digital marketing, and casinos or electronic gaming machines (Vizcaino et al., 2013). Our results suggest that in these environments, the pre-existing emotional valence of some cues may capture attention and thereby distort patterns of cue-outcome learning across the environment as a whole.

More broadly, our results fit within a growing body of research that studies the implications of cue-interaction effects (such as overshadowing) beyond the traditional associative learning paradigm. In recent years, this perspective has shed light on cue-interaction effects in social cognition and moral learning (FeldmanHall et al., 2017; Mata et al., 2021; Telga et al., 2023), and we likewise suggest that it may provide affective science with an indispensable framework for studying the role played by emotion in learning and decision making. An important next step in this research programme is to determine the extent to which the emotional valence of cues plays a role in canonical cue-interaction phenomena such as blocking, unblocking, overshadowing, and release from overshadowing (FeldmanHall & Dunsmoor, 2019; Lovibond et al., 2003).

## Supplementary Information

Below is the link to the electronic supplementary material.

Supplementary file1 (PDF 865 KB)

## Footnotes

1 The OASIS images were ‘Dog13’ and ‘Rainbow2’ (pleasant); ‘Animalcarcass5’ and ‘Fire7’ (unpleasant); and ‘Cups2’, ‘Storage1’, ‘Soldiers1’, and ‘Surgery5’ (neutral). Among the neutral images, we deliberately selected two low-arousal images and two high-arousal images.

## Additional Information

### Data Availability

The datasets generated and analysed during the current study are available in the Open Science Foundation repository at https://osf.io/q5x6f/.
