# Supplementary material for: Emotional Overshadowing: Pleasant and Unpleasant Cues Overshadow Neutral Cues in Human Associative Learning
Source: Affect Sci. 2024 Sep 7;5(3):222–31. doi: 10.1007/s42761-024-00270-0 (PMC11461368; doi:10.1007/s42761-024-00270-0)
Supplement: Supplementary file 1 — Supplementary file1 (PDF 865 KB) [file 42761_2024_270_MOESM1_ESM.pdf]

Supplementary Material for

Emotional overshadowing: pleasant and unpleasant cues overshadow neutral cues in human associative learning

Jianming Zhu[1], Angela Radulescu[2] & Daniel Bennett[1*]

[1] School of Psychological Sciences, Monash University, Melbourne, Australia

[2] Icahn School of Medicine at Mount Sinai, New York City, USA

* daniel.bennett@monash.edu

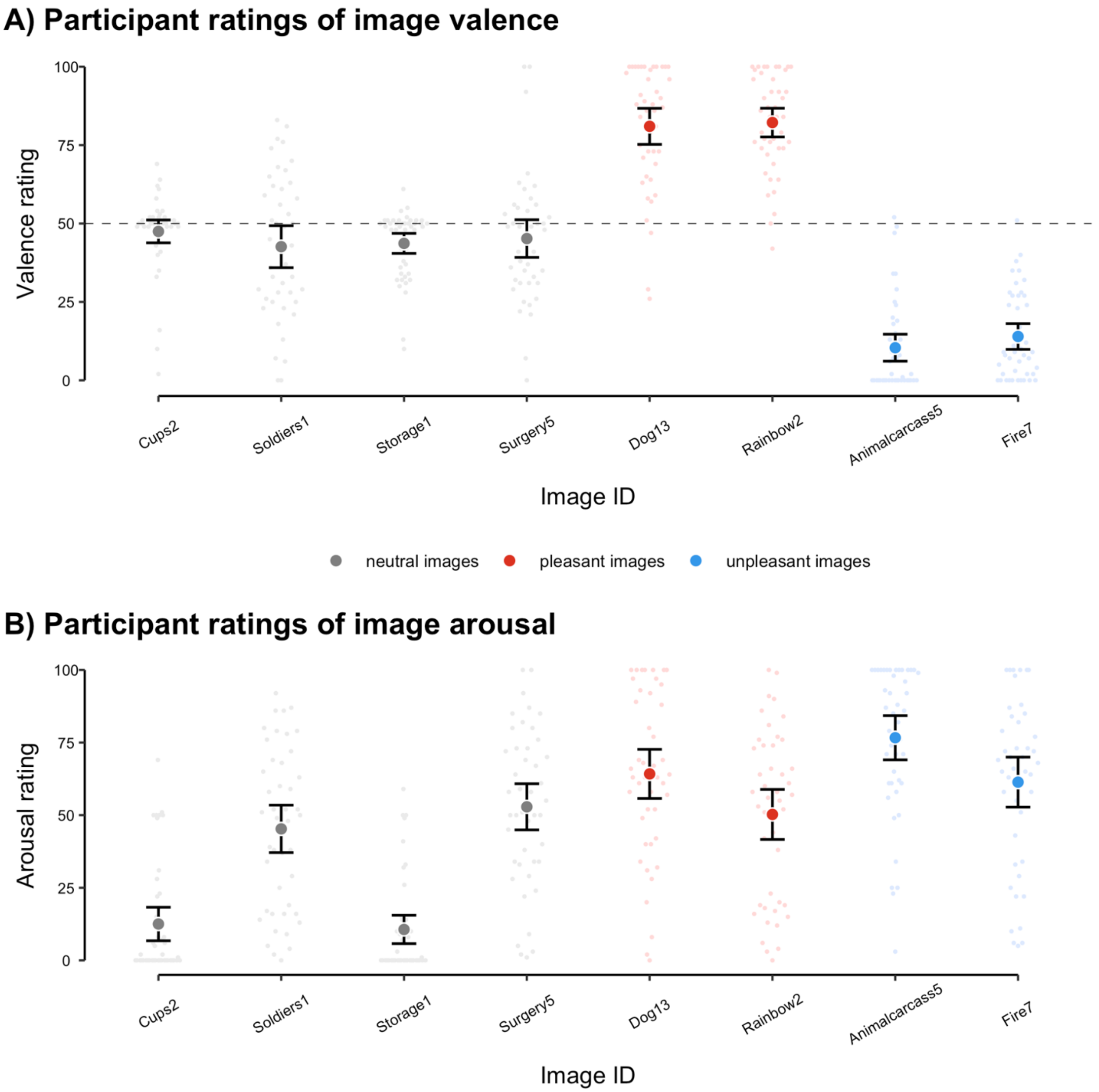

Supplementary Figure S1. Participant ratings of valence **(A)** and arousal **(B)** for each image in each category (grey: neutral-valence images; red: pleasant-valence images; blue: unpleasant-valence images) in the initial exploratory study. Foreground points and error bars denote group means (+/- 95% confidence interval); background points denote ratings from individual participants. The dashed horizontal line in (A) denotes neutral valence ("neither positive nor negative").

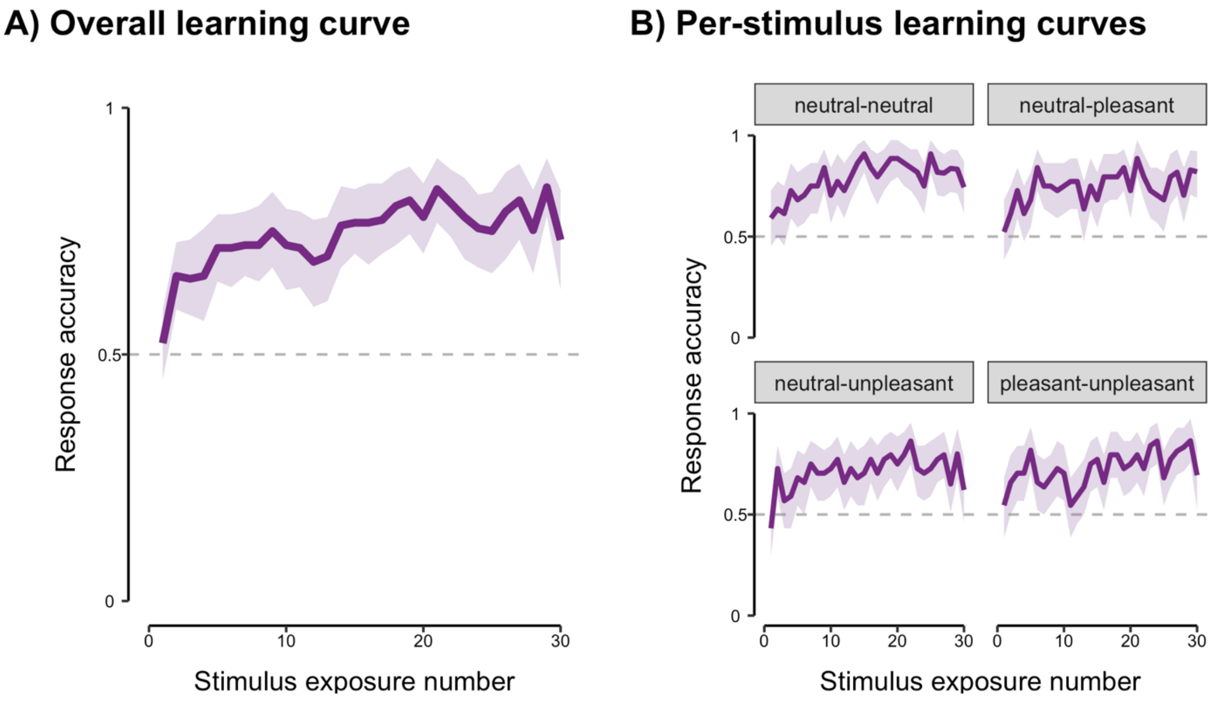

Supplementary Figure S2. Response accuracy as a function of stimulus exposure number during the learning phase of the probabilistic categorisation task for the initial exploratory study. Results are presented overall **(A)** and individually by stimulus type **(B)**. Data are mean response accuracy (with a correct response defined as predicting the most likely outcome for a stimulus) +/- 95% confidence interval. The dashed horizontal line denotes chance-level accuracy.

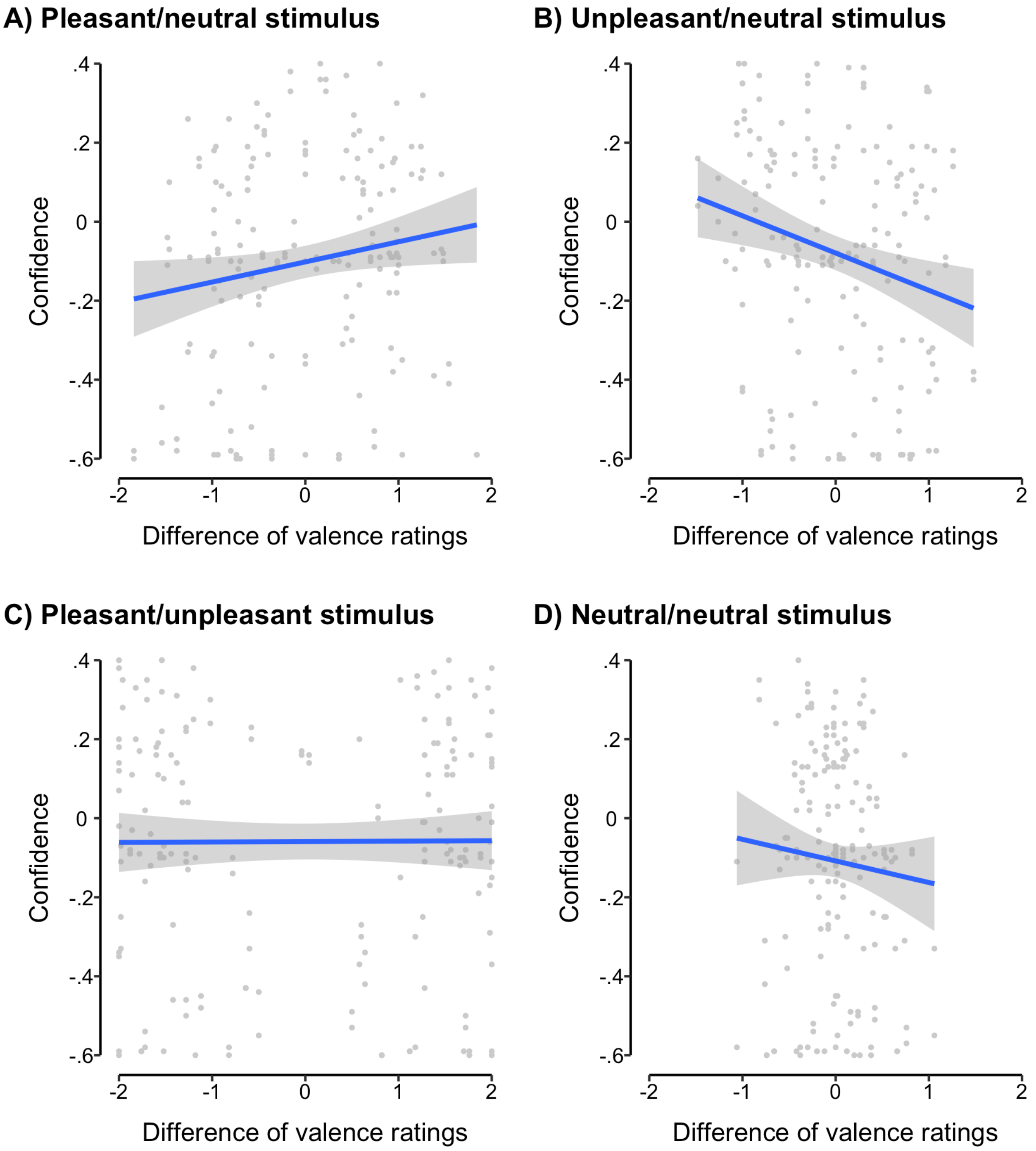

Supplementary Figure S3. The association between the difference in valence ratings between the two images in a stimulus (x-axis) and participants' confidence regarding reported cue-outcome associations (y-axis). There was a significant positive association between valence difference and confidence for the pleasant/neutral stimulus (A), a significant negative association for the unpleasant/neutral stimulus (B), and no significant association for either the pleasant/unpleasant stimulus (C) or the neutral/neutral stimulus (D). Regression lines indicate the linear association of best fit and its 95% confidence interval. Each background point represents one self-report by one participant. Data are presented for the initial exploratory study only.

Supplementary Table S1a. Overview, mixed-effects Bayesian regression of image valence ratings

**Fixed effects**	**Participant-wise random effects**
- Intercept<br>- Image type (reference-coded, *neutral* as reference)	- Random intercept<br>- Random slopes for:<br>- Image type

Note: valence ratings were standardised between -1 and 1 prior to analysis, such that a rating of 0 represents the mid-point of the rating scale.

Supplementary Table S1b. Coefficients for analysis of image valence ratings, exploratory study

**Coefficient**	***β* (SE)**	**95% Bayesian HDI**	
Intercept	-0.11 (0.03)	[-0.17, -0.04]	*
Image type (*pleasant*)	0.74 (0.05)	[0.63, 0.84]	*
Image type (*unpleasant*)	-0.65 (0.04)	[-0.74, -0.56]	*

Note: * denotes coefficients for which the Bayesian 95% HDI excludes zero

Supplementary Table S1c. Coefficients for analysis of image valence ratings, replication study

**Coefficient**	***β* (SE)**	**95% Bayesian HDI**	
Intercept	-0.07 (0.01)	[-0.09, -0.04]	*
Image type (*pleasant*)	0.69 (0.03)	[0.64, 0.74]	*
Image type (*unpleasant*)	-0.68 (0.02)	[-0.73, -0.63]	*

Note: * denotes coefficients for which the Bayesian 95% HDI excludes zero

Supplementary Table S2a. Overview, mixed-effects Bayesian regression of image arousal ratings

**Fixed effects**	**Participant-wise random effects**
- Intercept<br>- Image type (reference-coded, *neutral* as reference)	- Random intercept<br>- Random slopes for:<br>- Image type

Note: arousal ratings were standardised between -1 and 1 prior to analysis, such that a rating of 0 represents the mid-point of the rating scale.

Supplementary Table S2b. Coefficients for analysis of image arousal ratings, exploratory study

**Coefficient**	**β (SE)**	**95% Bayesian HDI**	
Intercept	-0.39 (0.05)	[-0.50, -0.29]	*
Image type (*pleasant*)	0.54 (0.08)	[0.39, 0.69]	*
Image type (*unpleasant*)	0.77 (0.08)	[0.63, 0.92]	*

Note: * denotes coefficients for which the Bayesian 95% HDI excludes zero

Supplementary Table S2c. Coefficients for analysis of image arousal ratings, replication study

**Coefficient**	**β (SE)**	**95% Bayesian HDI**	
Intercept	-0.42 (0.02)	[-0.46, -0.37]	*
Image type (*pleasant*)	0.55 (0.04)	[0.47, 0.62]	*
Image type (*unpleasant*)	0.75 (0.04)	[0.68, 0.83]	*

Note: * denotes coefficients for which the Bayesian 95% HDI excludes zero

Supplementary Table S3a. Overview, mixed-effects Bayesian logistic regression of learning data

Fixed effects	Participant-wise random effects
- Intercept<br>- Stimulus type (reference-coded with *neutral/neutral* as reference condition)<br>- Per-stimulus exposure number (*z*-scored)<br>- Stimulus type × exposure number	- Random intercept<br>- Random slopes for:<br>- Stimulus type<br>- Per-stimulus exposure number<br>- Stimulus type × exposure number

Note: The dependent variable was accuracy, coded as 1 if the participant predicted the most likely outcome shape for a given stimulus and 0 if they predicted the less likely shape.

Supplementary Table S3b. Coefficients for analysis of learning data, exploratory study

Coefficient	*β* (SE)	95% Bayesian HDI	
Intercept	1.92 (0.25)	[1.46, 2.41]	*
Stimulus type (*pleasant/neutral*)	0.10 (0.27)	[-0.40, 0.67]	
Stimulus type (*unpleasant/neutral*)	-0.52 (0.21)	[-0.92, -0.11]	*
Stimulus type (*pleasant/unpleasant*)	-0.16 (0.27)	[-0.68, 0.39]	
Exposure number	0.67 (0.14)	[0.39, 0.97]	*
Exposure number × stimulus type (*pleasant/neutral*)	0.14 (0.21)	[-0.24, 0.58]	
Exposure number × stimulus type (*unpleasant/neutral*)	-0.15 (0.14)	[-0.41, 0.14]	
Exposure number × stimulus type (*pleasant/unpleasant*)	0.09 (0.18)	[-0.23, 0.46]	

Note: * denotes coefficients for which the Bayesian 95% HDI excludes zero

Supplementary Table S3c. Coefficients for analysis of learning data, replication study

Coefficient	*β* (SE)	95% Bayesian HDI	
Intercept	1.99 (0.15)	[1.71, 2.29]	*
Stimulus type (*pleasant/neutral*)	0.54 (0.16)	[0.22, 0.87]	*
Stimulus type (*unpleasant/neutral*)	0.57 (0.16)	[0.26, 0.90]	*
Stimulus type (*pleasant/unpleasant*)	0.63 (0.18)	[0.29, 0.99]	*
Exposure number	0.92 (0.09)	[0.75, 1.11]	*
Exposure number × stimulus type (*pleasant/neutral*)	0.33 (0.12)	[0.11, 0.57]	*
Exposure number × stimulus type (*unpleasant/neutral*)	0.25 (0.12)	[0.03, 0.49]	*
Exposure number × stimulus type (*pleasant/unpleasant*)	0.29 (0.12)	[0.06, 0.53]	*

Note: * denotes coefficients for which the Bayesian 95% HDI excludes zero

Supplementary Table S4a. Overview, mixed-effects Bayesian regressions of learned contingencies (applies to all models reported in Tables S4-S7)

**Fixed effects**	**Participant-wise random effects**
- Intercept<br>- Queried shape (reference-coded with *square* as reference condition)<br>- Confidence rating for corresponding compound stimulus (i.e., for each simple cue, what was the confidence rating for the compound it was originally presented within?)<br>- Anxiety (GAD-7; *z*-scored)<br>- Valence difference (between this cue image and the other cue with which it was paired during learning)<br>- Arousal difference (between this cue image and the other cue with which it was paired during learning)<br>- Anxiety × Valence difference<br>- Anxiety × Arousal difference	- Random intercept<br>- Random slopes for:<br>- Queried shape<br>- Confidence rating for corresponding compound stimulus<br>- Valence difference<br>- Arousal difference

Note: - Valence and arousal differences were each based on participant-specific image ratings

Supplementary Table S4b. Analysis of cue images from pleasant/neutral stimulus, exploratory study

Coefficient	β (SE)	95% Bayesian HDI	
Intercept	-0.06 (0.03)	[-0.12, -0.01]	*
Queried shape	0.03 (0.02)	[-0.02, 0.07]	
Confidence rating for corresponding compound stimulus	0.57 (0.09)	[0.38, 0.75]	*
Anxiety	-0.03 (0.02)	[-0.07, 0.02]	
Valence difference between cues in compound	0.06 (0.02)	[0.02, 0.10]	*
Arousal difference between cues in compound	-0.01 (0.02)	[-0.05, 0.04]	
Anxiety × Valence difference	0.04 (0.02)	[0.001, 0.08]	*
Anxiety × Arousal difference	0.01 (0.02)	[-0.03, 0.06]	

Note: * denotes coefficients for which the Bayesian 95% HDI excludes zero

Supplementary Table S4c. Analysis of cue images from pleasant/neutral stimulus, replication study

Coefficient	β (SE)	95% Bayesian HDI	
Intercept	-0.03 (0.01)	[-0.06, -0.01]	*
Queried shape	0.01 (0.01)	[-0.01, 0.03]	
Confidence rating for corresponding compound stimulus	0.64 (0.04)	[0.55, 0.72]	*
Anxiety	-0.003 (0.01)	[-0.02, 0.01]	
Valence difference between cues in compound	0.02 (0.01)	[0.004, 0.04]	*
Arousal difference between cues in compound	-0.0004 (0.01)	[-0.02, 0.01]	
Anxiety × Valence difference	0.003 (0.01)	[-0.01, 0.02]	
Anxiety × Arousal difference	0.01 (0.01)	[-0.01, 0.02]	

Note: * denotes coefficients for which the Bayesian 95% HDI excludes zero

Supplementary Table S5a. Analysis of cue images from unpleasant/neutral stimulus, exploratory study

Coefficient	*β* (SE)	95% Bayesian HDI	
Intercept	-0.05 (0.02)	[-0.09, -0.01]	*
Queried shape	0.01 (0.02)	[-0.04, 0.05]	
Confidence rating for corresponding compound stimulus	0.64 (0.10)	[0.45, 0.84]	*
Anxiety	0.03 (0.02)	[-0.01, 0.07]	
Valence difference between cues in compound	-0.07 (0.03)	[-0.13, -0.01]	*
Arousal difference between cues in compound	0.03 (0.02)	[-0.01, 0.07]	
Anxiety × Valence difference	0.03 (0.03)	[-0.03, 0.09]	
Anxiety × Arousal difference	0.03 (0.02)	[-0.01, 0.06]	

Note: * denotes coefficients for which the Bayesian 95% HDI excludes zero

Supplementary Table S5b. Analysis of cue images from unpleasant/neutral stimulus, replication study

Coefficient	*β* (SE)	95% Bayesian HDI	
Intercept	-0.02 (0.01)	[-0.04, -0.01]	*
Queried shape	0.01 (0.01)	[-0.01, 0.03]	
Confidence rating for corresponding compound stimulus	0.63 (0.05)	[0.53, 0.74]	*
Anxiety	-0.02 (0.01)	[-0.03, .0001]	
Valence difference between cues in compound	-0.03 (0.01)	[-0.06, -0.001]	*
Arousal difference between cues in compound	0.01 (0.01)	[-0.02, 0.03]	
Anxiety × Valence difference	-0.01 (0.01)	[-0.03, 0.02]	
Anxiety × Arousal difference	-0.01 (0.01)	[-0.03, 0.01]	

Note: * denotes coefficients for which the Bayesian 95% HDI excludes zero

Supplementary Table S6a. Analysis of cue images from pleasant/unpleasant stimulus, exploratory study

Coefficient	β (SE)	95% Bayesian HDI	
Intercept	-0.02 (0.03)	[-0.09, -0.04]	*
Queried shape	0.001 (0.03)	[-0.05, 0.05]	
Confidence rating for corresponding compound stimulus	0.49 (0.14)	[0.21, 0.78]	*
Anxiety	-0.01 (0.03)	[-0.08, 0.06]	
Valence difference between cues in compound	0.004 (0.01)	[-0.01, 0.02]	
Arousal difference between cues in compound	0.02 (0.02)	[-0.02, 0.06]	
Anxiety × Valence difference	0.03 (0.01)	[0.01, 0.05]	*
Anxiety × Arousal difference	-0.01 (0.02)	[-0.05, 0.03]	

Note: * denotes coefficients for which the Bayesian 95% HDI excludes zero

Supplementary Table S6b. Analysis of cue images from pleasant/unpleasant stimulus, replication study

Coefficient	β (SE)	95% Bayesian HDI	
Intercept	-0.01 (0.01)	[-0.03, -0.01]	*
Queried shape	-0.01 (0.01)	[-0.03, 0.01]	
Confidence rating for corresponding compound stimulus	0.55 (0.06)	[0.44, 0.66]	*
Anxiety	0.01 (0.01)	[-0.01, 0.02]	
Valence difference between cues in compound	0.01 (0.01)	[-0.001, 0.01]	
Arousal difference between cues in compound	0.02 (0.01)	[-0.001, 0.04]	
Anxiety × Valence difference	0.004 (0.003)	[-0.01, 0.003]	
Anxiety × Arousal difference	-0.01 (0.01)	[-0.03, 0.01]	

Note: * denotes coefficients for which the Bayesian 95% HDI excludes zero

Supplementary Table S7a. Analysis of cue images from neutral/neutral stimulus, exploratory study

Coefficient	***β*** **(SE)**	**95% Bayesian HDI**	
Intercept	-0.06 (0.02)	[-0.11, -0.02]	*
Queried shape	0.02 (0.02)	[-0.02, 0.07]	
Confidence rating for corresponding compound stimulus	0.68 (0.08)	[0.53, 0.83]	*
Anxiety	0.01 (0.02)	[-0.03, 0.05]	
Valence difference between cues in compound	-0.05 (0.04)	[-0.14, 0.03]	
Arousal difference between cues in compound	-0.003 (0.02)	[-0.05, 0.05]	
Anxiety × Valence difference	0.05 (0.05)	[-0.06, 0.16]	
Anxiety × Arousal difference	0.0003 (0.02)	[-0.04, 0.05]	

Note: * denotes coefficients for which the Bayesian 95% HDI excludes zero

Supplementary Table S7b. Analysis of cue images from neutral/neutral stimulus, replication study

Coefficient	***β*** **(SE)**	**95% Bayesian HDI**	
Intercept	-0.05 (0.01)	[-0.08, -0.03]	*
Queried shape	-0.01 (0.01)	[-0.01, 0.03]	
Confidence rating for corresponding compound stimulus	0.60 (0.06)	[0.49, 0.72]	*
Anxiety	-0.01 (0.01)	[-0.03, 0.01]	
Valence difference between cues in compound	0.02 (0.02)	[-0.01, 0.06]	
Arousal difference between cues in compound	0.003 (0.01)	[-0.01, 0.02]	
Anxiety × Valence difference	0.02 (0.02)	[-0.02, 0.06]	
Anxiety × Arousal difference	-0.0001 (0.01)	[-0.02, 0.02]	

Note: * denotes coefficients for which the Bayesian 95% HDI excludes zero

Supplementary Table S8a. Coefficients for aggregate regression analysis, initial exploratory study

**Coefficient**	***β*** **(SE)**	**95% Bayesian HDI**	
Intercept	-0.05 (0.02)	[-0.09, -0.01]	*
Queried shape	0.01 (0.01)	[-0.01, 0.04]	
Confidence rating for corresponding compound stimulus	0.50 (0.06)	[0.38, 0.63]	*
Anxiety	0.004 (0.02)	[-0.04, 0.04]	
Valence difference between cues in compound	-0.003 (0.01)	[-0.02, 0.01]	
Arousal difference between cues in compound	-0.004 (0.01)	[-0.03, 0.02]	
Difference in absolute valence between cues	0.08 (0.02)	[0.03, 0.12]	*
Anxiety × valence difference	0.03 (0.01)	[0.01, 0.04]	*
Anxiety × arousal difference	0.01 (0.01)	[-0.02, 0.03]	
Anxiety × difference in absolute valence	0.03 (0.02)	[-0.01, 0.08]	

Note: * denotes coefficients for which the Bayesian 95% HDI excludes zero

Supplementary Table S8b. Coefficients for aggregate regression analysis, replication study

**Coefficient**	***β*** **(SE)**	**95% Bayesian HDI**	
Intercept	-0.03 (0.01)	[-0.05, -0.02]	*
Queried shape	0.002 (0.01)	[-0.01, 0.02]	
Confidence rating for corresponding compound stimulus	0.51 (0.03)	[0.44, 0.58]	*
Anxiety	-0.01 (0.01)	[-0.03, 0.01]	
Valence difference between cues in compound	-0.001 (0.01)	[-0.01, 0.01]	
Arousal difference between cues in compound	0.01 (0.01)	[-0.004, 0.02]	
Difference in absolute valence between cues	0.02 (0.01)	[0.003, 0.03]	*
Anxiety × valence difference	-0.002 (0.01)	[-0.01, 0.01]	
Anxiety × arousal difference	-0.001 (0.01)	[-0.01, 0.01]	
Anxiety × difference in absolute valence	0.01 (0.01)	[-0.01, 0.02]	

Note: * denotes coefficients for which the Bayesian 95% HDI excludes zero
